# Author's reply

**DOI:** 10.1192/bjb.2021.121

**Published:** 2022-02

**Authors:** Lucy Johnstone

**Affiliations:** Consultant Clinical Psychologist and Independent Trainer. Email: LucyJohnstone16@blueyonder.co.uk

I do indeed agree with the statements in Professor House's final paragraph and with several of his other points, for example, that there are many vested interests in the debate about public mental health, and that we can see the term ‘mental health’ itself as both effect and cause of the individualisation of societal problems. Indeed, it is that individualisation – not, as he seems to assume, the psychiatric profession itself – that my critique is aimed at. I have always argued that all professions, including my own, need to be aware of the limitations and potential harms of their theories and practices. That is why I raised concerns not just about over prescribing, but about ‘formal psychological interventions [which may be] unnecessary for most and can actually be harmful if implemented too early.’

I find Professor House's final phrase ‘…a muddled polemic animated as much as anything else by anti-psychiatry sentiment’ the most worrying part of his response. This kind of language suggests that he has moved beyond rational and evidence-based argument, into *ad hominem* dismissal. It invites a fight rather than a debate, and since I do not identify as ‘anti-psychiatry’ (whatever that means) I have no desire to take up such a challenge. I will simply observe that the areas in which I take a different position from him are fundamental, legitimate and increasingly common. For example, clinical psychologists’ professional guidelines on formulation state that it is ‘not premised on a functional psychiatric diagnosis’.^1^ Professor House is free to use the term differently but not to simply rule other definitions out of court. Yes, we need to offer immediate help to individuals as well as addressing adversities, but that help does not have to be based on unproven medical assumptions about the nature and origins of their distress. Yes, there are social causal factors and unclear boundaries in some physical health conditions, but no one is arguing that diabetes is a mental health problem; common sense tells us that this analogy doesn't work, despite the claims of anti-stigma campaigns and some professionals. And so on.

In 2017, a United Nations report noted ‘The urgent need to…target social determinants and abandon the predominant medical model that seeks to cure individuals by targeting “disorders”’ and recommended that ‘Mental health policies should address the “power imbalance” rather than “chemical imbalance”’.^2^ Rather than allowing ourselves to be distracted by attempts to defend a failed paradigm, we all urgently need to work towards this future.
